# Comparative analysis of antioxidant status in diabetic patients with and without insulin resistance

**DOI:** 10.25122/jml-2023-0079

**Published:** 2023-09

**Authors:** Suad Lateef Ibrahim, Nadia Khadam Jawad Al-Dawah, Muna Abdulridha Al-Barqaawi

**Affiliations:** 1 Department of Clinical Laboratory Sciences, Faculty of Pharmacy, University of Kufa, Kufa, Iraq; 2 Department of Physiology and Pharmacology, Faculty of Veterinary Medicine, University of Kufa, Kufa, Iraq; 3 Department of Biochemistry, Faculty of Medicine, University of Kufa, Kufa, Iraq

**Keywords:** diabetes mellitus, glutathione, malondialdehyde, uric acid, lipid profile

## Abstract

Diabetes mellitus (DM) is a metabolic disorder characterized by insulin resistance, where hyperglycemia is believed to trigger oxidative stress, contributing to insulin function impairment. This study aimed to assess and compare levels of malondialdehyde (MDA), glutathione (GSH), and uric acid in diabetic patients with and without insulin resistance and to assess the correlation with fasting blood sugar (FBS) and lipid profiles. Significant variations were found in MDA, uric acid, and GSH levels between insulin-resistant and non-resistant diabetic groups (p<0.0001). FBS, hemoglobin A1C (HbA1C), insulin activity, and Homeostatic Model Assessment for Insulin Resistance (HOMA-IR) significantly differed between the groups (p<0.0001). Total cholesterol, triglyceride, high-density lipoprotein (HDL), very-low-density lipoprotein (VLDL), and low-density lipoprotein-cholesterol (LDL-C) concentrations were higher in the insulin resistance group than the non-insulin resistance group (p<0.0001). Uric acid also exhibited a significant correlation (p<0.01) with LDL levels, while HDL levels showed a negative correlation with both MDA and uric acid (p<0.001). Diabetes mellitus, characterized by chronic hyperglycemia, may play a role in the development of oxidative stress. This oxidative stress is a significant factor that could potentially lead to the onset of insulin resistance, a condition strongly associated with dyslipidemia. The results of this study indicate that the decrease in GSH levels and the increase in MDA and uric acid levels are particularly noteworthy in the context of insulin resistance among patients with diabetes.

## INTRODUCTION

Diabetes mellitus (DM) is a metabolic disorder characterized by elevated blood sugar levels resulting from defects in insulin action, secretion, or both [1]. Diabetes is one of the most important diseases globally, with projections indicating that almost 69% of adults could be affected by 2030 [2]. Oxidative stress (OS) occurs when the free radical generation rate is improved in the antioxidant system, causing the free radical toxic effects [3]. Numerous studies have suggested that OS has a critical role in the pathogenesis of DM and its complications along with insulin resistance development [4]. Elevated blood sugar levels contribute to increased OS, compromising insulin action and secretion. Moreover, patients with diabetes mellitus often exhibit compromised antioxidant mechanisms, leading to heightened oxidative stress [5, 6]. Consequently, studies exploring dietary antioxidants like vitamins to mitigate the impact of diabetes-related complications have gained attention [7, 8]. This study aimed to assess and compare the serum levels of malondialdehyde (MDA), glutathione (GSH), and uric acid in diabetic patients with and without insulin resistance while also investigating the potential correlation between fasting blood sugar (FBS) and lipid profiles with these antioxidant parameters.

## MATERIAL AND METHODS

### Study design and data collection

This study enrolled 170 diabetic patients diagnosed with non-insulin-dependent diabetes mellitus (NIDDM) (type 2 DM) from the Center of Diabetics at AL-Sadder Teaching Hospital between July 2011 and January 2012. To facilitate comparisons in the biochemical changes associated with diabetes, 50 patients diagnosed with type 1 diabetes were also included in the study as a control group. Demographic data, including age, sex, weight, height, and disease duration, were collected through a structured questionnaire.

### Sample collection

Blood samples were collected from patients using venipuncture. Collected samples were allowed to clot at 37°C and subsequently centrifuged at 3000 xg for 10 minutes. The resulting sera were separated and stored in disposable serum tubes at -17°C until analysis. Serum was utilized to assess insulin activity, MDA, GSH, uric acid levels, and lipid profiles in patients and the control groups.

### Methods

Insulin activity was calculated using the homeostasis model assessment (HOMA) formula: (glucose × insulin)/405 [9]. The measurement of malondialdehyde involved the modified Talat technique, wherein MDA reacts with thiobarbituric acid (TBA) to form MDA-TBA2 [10]. GSH levels were determined through a colorimetric method involving the reduction of 5,5-dithiobis (2-nitrobenzoic acid) (DTNB) by the sulfhydryl group of GSH [10]. Serum uric acid levels were quantified using an enzymatic Uricase procedure (Biolabo kit, Cat. No. 80001). Cholesterol levels were measured via enzymatic hydrolysis and oxidation, with phenol and peroxidase generating the indicator quinonemine from hydrogen peroxide and 4-amino antipyrin [11]. For triglyceride quantification, an enzymatic colorimetric method was employed [12]. Liquid high-density lipoprotein cholesterol (HDL-C) precipitant was utilized for measuring HDL-C [13]. Lastly, the serum level of low-density lipoprotein cholesterol (LDL-C) was estimated using Friedewald's empirical formula: Total Cholesterol - HDL-C - (TG/5) = LDL-C [13].

### Statistical analysis

The results were expressed as mean ± SD, and categorical variables were presented as frequency (N). Student's t-test was used to investigate the association of oxidative stress marker and serum lipid profile in the patient group compared to the control group. Additionally, linear regression analysis was conducted to assess the impact of various disease-related parameters on oxidative stress levels and serum lipid profiles. A significance level of p<0.05 was considered statistically significant. The SPSS version 25.0 software (IBM SPSS, Chicago, IL, USA) was used for all statistical analyses.

## RESULTS

There were no significant differences in age and BMI between non-insulin resistance and diabetic patients with insulin resistance. However, a significant difference was observed in the duration of the disease (p<0.001; Table 1). The levels of lipid peroxidation (MDA), uric acid, and GSH were significantly different between diabetic patients with and without insulin resistance (p<0.0001; Table 1). Among the measured parameters, fasting blood glucose levels, hemoglobin A1C, insulin activity, and Homeostatic Model Assessment for Insulin Resistance (HOMA-IR) were significantly different between the two groups, with higher values in diabetic patients with insulin resistance (p<0.0001; Table 1). Moreover, the total cholesterol, triglycerides, HDL, VLDL, and LDL-C concentrations were significantly higher in the group with insulin resistance than in the non-insulin resistance group (p<0.0001; Table 1). Table 2 shows that there was a statistically significant inverse correlation between GSH levels, total cholesterol, triglycerides, and VLDL levels (p<0.05, p<0.01, and p<0.05, respectively). Additionally, a positive correlation between blood GSH levels and HDL levels was observed in the insulin resistance group (p<0.05). Moreover, a regression analysis demonstrated significant relationships between MDA and uric acid with total cholesterol, triglycerides, and VLDL levels (p<0.001). In terms of lipid profile, a significant correlation was observed between uric acid and LDL cholesterol (p<0.01), while HDL cholesterol had a negative association with both MDA and uric acid (p<0.001) (Table 2).

**Figure 1:**
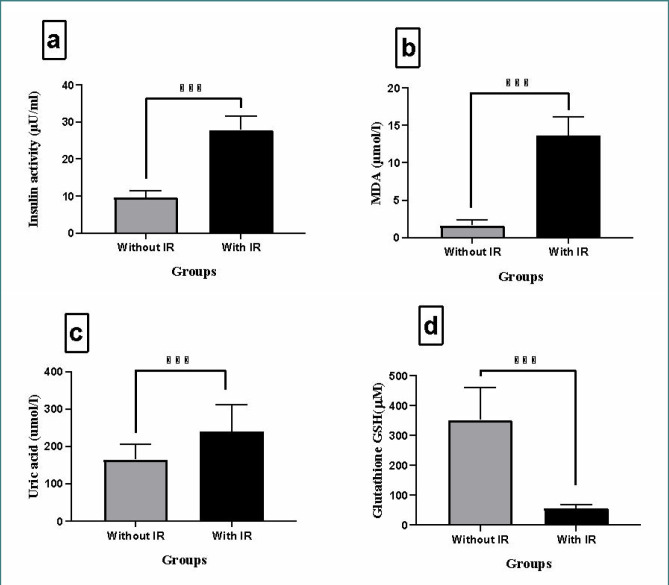
Comparison of insulin-resistant (IR) and non-insulin-resistant (non-IR) patient groups. The levels of insulin activity (a), malondialdehyde (MDA) (b), uric acid (c), and glutathione (GSH) (d) were compared between patients with insulin resistance and those without highlighting differences between the groups. Results are presented as means ± SD (n=3). Significant differences are indicated (p<0.001 paired t-test).

**Figure 2:**
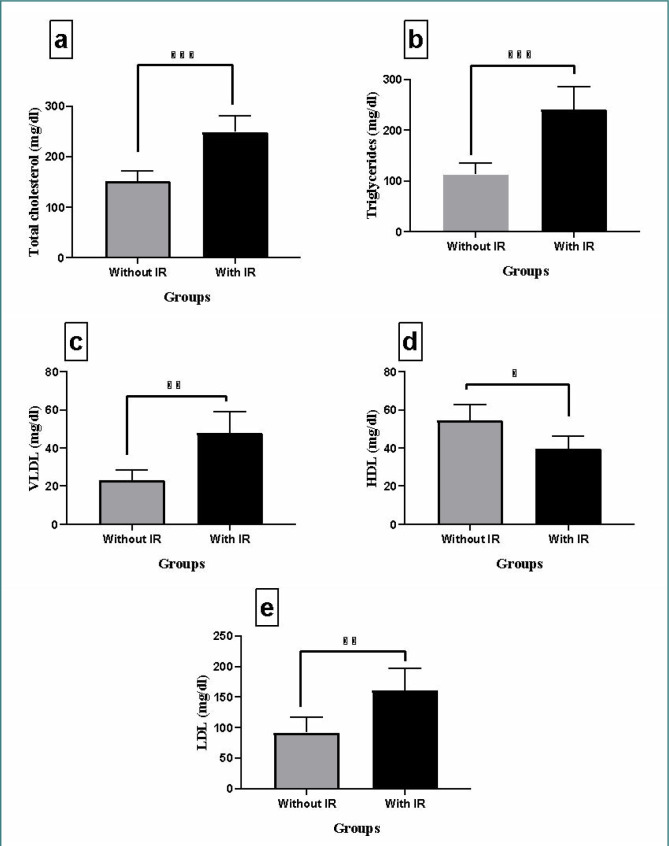
Comparison of insulin-resistant (IR) and non-insulin-resistant (non-IR) patient groups. The levels of total cholesterol (a), triglycerides (b), very-low-density lipoprotein (c), high-density lipoprotein (d), and low-density lipoprotein-cholesterol (e) were compared between patients with insulin resistance and those without to illustrate the differences between the groups. Results are presented as means ± SD (n=3). Significant differences are indicated (p<0.05, p<0.01, p<0.001 paired t-test).

We assessed clinical parameters, including insulin activity, GSH, MDA, uric acid, and lipid profile, across subjects in the non-insulin resistance group. The results of this analysis are depicted in Figure 1 (A-D) and Figure 2 (A-E). Figure 1 indicates a significant (p<0.001) increase in mean insulin activity, MDA, and uric acid levels among patients with insulin resistance compared to those without (p-value<0.001). GSH levels were significantly elevated (p<0.001) in the non-insulin resistance group (control). There were significant differences between insulin-resistant patients and non-insulin resistant group (control) (p<0.001, p<0.001, p<0.01, p<0.05, and p<0.01) for total cholesterol, triglyceride, VLDL, HDL, and LDL cholesterol levels, respectively, as illustrated in Figure 2 (A-E).

**Table 1:** Clinical characteristics of participants

Variables	Non-insulin resistance group	Insulin resistance group	p-value
**Number**	50	120	-
**Sex, male/female**	22/28	64/56	-
**Age, year**	44.96±9.10	46.96±15.7	N.S
**Body mass index (BMI)**	23.10±2.35	24.76±6.37	N.S
**Duration, year**	2.08±0.97	8.87±3.93	<0.0001*#
**Fasting blood sugar (FBS)**	80.24±7.00	214.90±19.45	<0.0001*#
**Hemoglobin A1C (HbA1C )**	6.08±0.26	8.22±0.96	<0.0001*#
**Insulin (µU/ml)**	9.62±1.86	27.87±3.59	<0.0001*#
**Insulin resistance (HOMA-IR)**	3.28±1.21	5.3±2.28	<0.0001*#
**Malondialdehyde (µmol/l)**	1.66±0.71	13.70±2.41	<0.0001*#
**Glutathione (µM)**	352.3±107.3	56.06±10.68	<0.0001*#
**Uric acid (umol/l)**	165.4±39.26	240.00±71.47	<0.0001*#
**Total cholesterol(mg/dl)**	152.30±20.15	249.30±31.55	<0.0001*#
**Triglyceride(mg/dl)**	113.10±21.91	241.6±43.26	<0.0001*#
**High-density lipoprotein (mg/dl)**	54.59±8.61	39.68±6.57	<0.0001*#
**Very-low-density lipoprotein (mg/dl)**	23.30±5.08	47.49±11.20	<0.0001*#
**Low-density lipoprotein-cholesterol (mg/dl)**	92.08±24.76	161.30±35.25	<0.0001*#

* Significant results; #Students t-test; n.s: not applicable

**Table 2:** Linear regression analysis between GSH, MDA, uric acid and lipid profile in a group with insulin resistance

Parameters	Total cholesterol (mg/dl)	Triglycerides (mg/dl)	Very-low-density lipoprotein (mg/dl)	High-density lipoprotein (mg/dl)	Low-density lipoprotein-cholesterol (mg/dl)
**Glutathione (µM)**	-0.21^a^	-0.25^b^	-0.24^b^	0.22^a^	0.13^f^
**Malondialdehyde (µmol/l)**	0.88^c^	0.85^c^	0.87^c^	-0.88^c^	0.12^f^
**Uric acid (umol/l)**	0.72^c^	0.68^c^	0.70^c^	-0.74^c^	0.21^a^

a: p<0.05; b: p<0.01; c: p<0.001; f: not significant

## DISCUSSION

Diabetes mellitus is characterized by elevated blood glucose levels and associated glucose and lipid peroxidation changes. Oxidative stress induced by hyperglycemia in diabetes has been implicated in impairing insulin signaling, contributing to the development of insulin resistance. The findings of this study support the role of oxidative stress in the complex etiology of insulin resistance in diabetes. The results revealed a significant increase in insulin resistance (HOMA-IR) in diabetic patients with a duration of diabetes ≥8 years compared to non-insulin-resistant patients, suggesting a potential link between persistent hyperglycemia and the development of insulin resistance. Aging is known to contribute to reduced lean body mass, which can further exacerbate insulin resistance [14]. The present study corroborates this by demonstrating that older age and higher body fat levels are associated with insulin resistance.

In this study, we examined the serum levels of the antioxidant enzyme GSH, the lipid peroxidation by-product MDA, and uric acid in patients with diabetes mellitus. We further correlated these serum values with insulin activity and lipid peroxidation. Almost 120/170 diabetic patients in our study had insulin resistance as a complication of diabetics that was consequently associated with decreased levels of antioxidant GSH in the serum of patients with IR compared to those without insulin resistance. This result is consistent with findings from a separate study where the author highlighted that oxidative stress can impact antioxidant levels, including GSH, leading to their decline [15]. Moreover, another study demonstrated a link between reduced glutathione levels and the development of serious conditions among individuals with type II diabetes [16].

In our investigation, we observed an elevation in the levels of MDA, a marker of lipid peroxidation, in the serum of diabetic patients with insulin resistance (IR) compared to non-IR patients. This finding aligns with a study conducted by Choosong *et al*., which reported similarly increased MDA levels and their correlation with insulin resistance in individuals with prediabetes [17]. This observation suggests the existence of disruptions between protective mechanisms like glutathione and the generation of reactive oxygen species (ROS), thereby augmenting the creation of oxidative stress. This means that some disorders related to oxidative stress are usually linked with the overproduction of ROS and/or diminished levels of antioxidant enzyme activities, resulting in insulin resistance [18].

The relationship between insulin resistance and uric acid levels has been previously established [19]. Our study also observed a connection between insulin resistance and elevated serum uric acid levels. A study by Zhi *et al*. [19] supported this finding, demonstrating that high uric acid levels can impede insulin signaling, potentially inducing insulin resistance in both in vitro and in vivo cardiomyocyte models.

Our findings are consistent with previous studies [20], which found that the levels of uric acid were elevated in patients with Type 2 diabetes mellitus (TD2M), a condition characterized by insulin resistance and impaired glucose tolerance [20]. This can be attributed to the elevation in purine biosynthesis, driven by increased activity of the hexose monophosphate pathway shunt. This elevation in purine biosynthesis could theoretically be linked to conditions associated with insulin resistance and/or hyperinsulinemia. In this context, the heightened flow of glucose-6-phosphate through the hexose monophosphate pathway shunt, stemming from glycolytic pathway impairment, has been proposed to explain uric acid elevation in cases of impaired glucose tolerance. This concept might also encompass excessive carbohydrate intake and increased lipogenesis in the presence of insulin excess [20].

Previous studies have demonstrated a correlation between abnormal glucose levels and disrupted lipid profiles, particularly marked by decreased high-density lipoprotein (HDL) levels, indicative of severe insulin resistance. The results revealed that diabetic patients with dyslipidemia had higher HOMA-IR, suggesting the presence of severe insulin resistance and damage compensatory response of β cells to insulin resistance. These outcomes are supported by other studies, which indicated that hyperglycemia and hyperlipidemia elevate reactive oxygen species production and oxidative damage, leading to insulin resistance and many problems, such as energy metabolism and vascular complications [15]. Similar studies found low high-density lipoprotein levels associated with hyperinsulinemia or insulin resistance. These significant variations are linked with insulin resistance syndrome, leading to increased triglycerides and decreased HDL-C levels. So, in dyslipidemia, using the lipoprotein concentration ratios is associated with insulin resistance and increased cardiovascular disease (CVD) risk conditions [21].

## CONCLUSION

**Figure 3:**
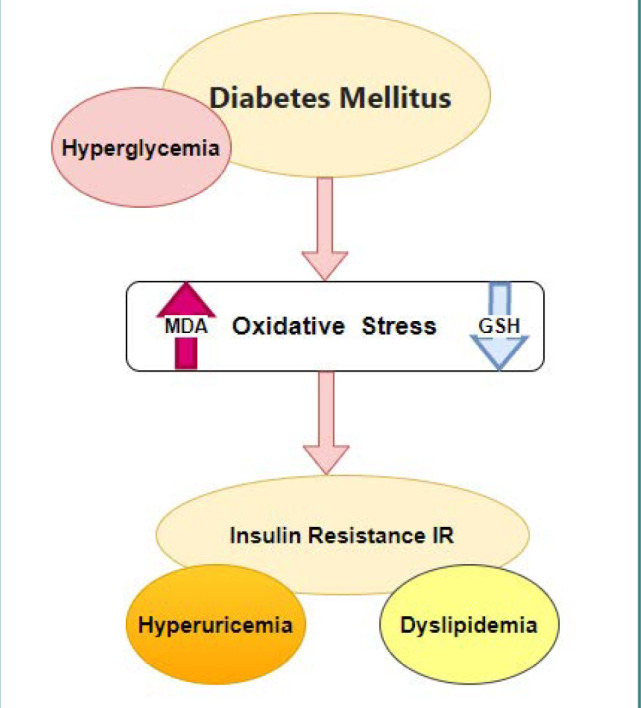
Proposed model of insulin resistance induction in patients with diabetes

Chronic hyperglycemia, a symptom of diabetes mellitus, may contribute to the onset of induced oxidative stress. Oxidative stress is a major issue that might lead to insulin resistance, which has a strong correlation with dyslipidemia. Our results strongly suggest that reduced GSH levels, elevated MDA levels – a marker of lipid peroxidation, and heightened uric acid levels play a crucial role in the context of insulin resistance in individuals with diabetes, as summarized in Figure 3.
